# Correlational Insights into Attention-Deficit/Hyperactivity Disorder in Lebanon

**DOI:** 10.3390/ijerph21081027

**Published:** 2024-08-05

**Authors:** Melyssa Assaf, Melissa Rouphael, Sarah Bou Sader Nehme, Michel Soufia, Abbas Alameddine, Souheil Hallit, Marc Landry, Tania Bitar, Walid Hleihel

**Affiliations:** 1 Department of Biology, Faculty of Arts and Sciences, Holy Spirit University of Kaslik, Jounieh P.O. Box 446, Lebanon; 2 UMR Inserm 1253 Ibrain, Université de Tours, 37032 Tours, France; 3 CNRS, Institute of Neurodegenerative Diseases, IMN, University of Bordeaux, UMR 5293, 33000 Bordeaux, France; 4 School of Medicine and Medical Sciences, Holy Spirit University of Kaslik, Jounieh P.O. Box 446, Lebanon; 5 North Autism Center (NAC), Zgharta 1304, Lebanon; 6 Department of Psychiatry, Hôtel-Dieu de France Hospital, A. Naccache Avenue—Achrafieh 1100, Beirut 166830, Lebanon; 7 Psychology Department, College of Humanities, Effat University, Jeddah 21478, Saudi Arabia; 8 Applied Science Research Center, Applied Science Private University, Amman 11937, Jordan

**Keywords:** attention-deficit/hyperactivity disorder, prenatal anemia, stress, neonatal jaundice, breastfeeding, familial ADHD history

## Abstract

Attention-Deficit/Hyperactivity Disorder (ADHD), a prevalent childhood neurodevelopmental disorder with complex etiology involving genetic and environmental factors, causes impairments across various life domains and substantial social and economic burden. Identifying correlates to prevent its onset and decrease its incidence is crucial. To our knowledge, our study represents the first case–control investigation of Lebanese ADHD patients to explore potential correlations between familial, maternal, and child health variables and ADHD to enhance understanding of its etiology and aid in prevention efforts. We recruited 61 Lebanese ADHD patients and 58 matched controls aged 6–24 years from all districts of Lebanon. The data to analyze were collected using a questionnaire. We employed statistical tests, including the independent samples *t*-test and the Chi-square test or Fisher’s exact test. We conducted a multivariate logistic regression analysis to identify the statistically significant factors explaining ADHD likelihood. We observed male predominance (68.9%) among patients. Maternal anemia during pregnancy (OR = 3.654; 95% CI [1.158–11.529]), maternal self-reported stress during pregnancy (OR = 3.268; 95% CI [1.263–8.456]), neonatal jaundice (OR = 5.020; 95% CI [1.438–17.532]), and familial history of ADHD (OR = 12.033; 95% CI [2.950–49.072]) were significantly associated with increased odds of the disorder. On the other hand, breastfeeding (OR = 0.263; 95% CI [0.092–0.757]) was identified as a protective factor against ADHD. This pilot study shed light on risk and protective factors associated with ADHD in the Lebanese population. The results are relevant, as some identified correlates could be avoidable. Further rigorous investigation is required to expand upon the observed correlations and to assist in early detection, prevention, and intervention strategies targeting ADHD.

## 1. Introduction

Attention-Deficit/Hyperactivity Disorder (ADHD) is a complex neurodevelopmental disorder characterized by impairing symptoms of inattention and/or hyperactivity–impulsivity [1]. Typically identified in early school years, ADHD is one of the most common childhood disorders that may persist into adolescence and adulthood [1]. Diagnosis, according to the *Diagnostic and Statistical Manual of Mental Disorders, 5th Edition* (DSM-5), requires symptom manifestation before the age of twelve, accompanied by substantial impairments in daily functioning [1]. The average age at diagnosis is reported to be around seven years [2,3].

ADHD often coexists with various conditions such as learning disorders, depression, anxiety, bipolar disorder, conduct disorder, oppositional defiant disorder, and substance use disorders [4,5]. Individuals with the disorder face multiple functional impairments, including academic and occupational failure, criminal tendencies, divorce, social rejection, suicide, injuries due to accidents, and premature death [5,6]. 

Globally, ADHD’s prevalence in children is estimated at 5% [1], while persistent adult ADHD is reported at approximately 2.58% [7]. Despite affecting both genders, males are diagnosed at a higher rate than females, with a sex ratio ranging from 2:1 to 10:1, attributed to variations in disorder manifestation between genders [8]. In Lebanon, research on the prevalence of ADHD is limited. According to Richa et al.’s 2014 epidemiological study, the reported prevalence of ADHD among school children across all regions was estimated at 8% [9]. Additionally, Ghossoub et al.’s 2017 study reported a prevalence of 10.2% among adolescents exclusively in the Beirut region [10]. Concerning the prevalence among Lebanese adults, as of now, no existing studies have explored this aspect within this demographic. 

The etiology of ADHD is not completely understood, yet it is regarded as multifactorial. Genetic predisposition was first proposed based on family, twin, and adoption studies, revealing a high heritability rate of approximately 74% [6]. Subsequently, research has identified numerous ADHD candidate genes associated primarily with the synthesis and transmission of monoamine neurotransmitters and implicated in neuronal proliferation, survival, maturation, and synaptic function [6]. The sub-100% heritability in ADHD suggests substantial environmental impact, especially during the vulnerable prenatal and postnatal stages when exposure to harmful agents can affect the epigenome and disrupt gene expression critical to central nervous system development, heightening the risk of neurodevelopmental disorders [11]. Within this context, several environmental factors have been suggested as potential contributors to ADHD. Some of the reported factors associated with the mother during the prenatal period encompass previous abortion, the use of assisted reproductive technologies, pre-pregnancy obesity, excessive gestational weight gain, gestational diabetes, pre-eclampsia, infections, physical trauma, and smoking [12,13,14,15,16]. Prior research has indicated a correlation between maternal anemia during pregnancy and an elevated risk of neurodevelopmental disorders, including ADHD [17]. Moreover, previous studies have established an association between prenatal stress and the manifestation of ADHD symptoms in the offspring [18,19]. The correlation between maternal stress and altered infant cognitive development has also been documented [20]. As for the perinatal and neonatal periods, research described factors such as cesarian section, low birth weight, lactation type, and head trauma as possible contributors to the disorder [14,21]. Additionally, being born preterm is associated with a risk of developing ADHD, as reported in several studies [22,23]. Moreover, other studies have shown that jaundiced neonates were at higher risk for developing ADHD during their growth period than non-jaundiced controls [24,25]. Some studies also pointed to the influence of an adverse psychosocial environment, such as physical and emotional abuse, on increasing the susceptibility to ADHD [26]. 

Until now, there has been no cure for ADHD. Current pharmacological and non-pharmacological interventions can help address the symptoms of the disorder to alleviate their adverse effects on academic and social functioning [27]. Furthermore, the economic implications associated with raising children with ADHD are markedly higher when compared to those without the disorder [28]. This imposes a significant burden on both individuals and their families, highlighting ADHD as a matter of considerable health relevance and underscoring the imperative for effective prevention and intervention programs to prevent the onset of symptoms and their deleterious long-term consequences.

While existing research exploring determinants of ADHD stems predominantly from Western contexts, the necessity for national and regional studies becomes apparent due to the potential influence of cultural and contextual factors on the disorder’s development [29]. Motivated by these considerations, we conducted a first case–control study in Lebanon to replicate existing findings and check whether previously reported ADHD risk factors held within a representative Middle-Eastern Lebanese sample. Our investigation focuses on variables related to maternal, child, and familial characteristics, with the ultimate goal of identifying modifiable correlates that could enhance our understanding of ADHD’s etiology and establish a foundation for informed interventions capable of attenuating the risk for this prevalent disorder.

## 2. Materials and Methods

### 2.1. Sample Size Calculation

The minimal sample size needed for this study was calculated using Epi-info software, resulting in 52 cases and 52 controls. This calculation relied on a power of 80%, a confidence level of 95%, and a percentage of being born via a C-section delivery of 35.5% among controls and 62.8% among cases, as derived from previous research [30].

### 2.2. Study Population, Recruitment, and Data Collection

A case–control study was conducted from February to November 2023 on 119 Lebanese individuals aged 6–24 years old encompassing all districts of Lebanon (North, Bekaa, Mount Lebanon, Beirut, and South). As the stability of ADHD diagnosis during the preschool period is uncertain [31], only individuals aged 6 years and older were included in this study. Cases consisted of 61 individuals diagnosed with ADHD, recruited from specialized organizations and private clinics. Their assessment was conducted using the judgment of experienced clinicians. Criteria for diagnosis were based on DSM-5 guidelines. Diagnosis involved clinical interviews with patients and their families. For children, parents completed the ADHD Rating Scale, while teachers filled out the Connors and Vanderbilt Rating Scales. For adults, the Adult ADHD Self-Report Scale and the Wender Utah Rating Scale were used. Additionally, psychometric testing included the Test of Variables of Attention (T.O.V.A.), with speech and psychomotor assessments conducted in some cases. Controls included 58 typically developing individuals of the same gender, age, and districts, selected from regular schools. Information for both cases and controls was derived from mothers. To ensure typical development, mothers of potential controls were provided with the primary signs and symptoms of ADHD. They were then asked whether their children had been diagnosed or were suspected to have ADHD or any other psychiatric illness. Data were collected through telephone interviews using a questionnaire prepared in the Arabic language. Parents of both cases and controls were contacted by the treating team and the school’s administration to inform them about the study and obtain their consent for sharing contact information with the research team. Upon receiving lists of willing participants, the research team called the mothers to explain the study’s scope and obtain oral consent before proceeding with the interviews. On average, the questionnaire took approximately 15 to 20 min to complete. 

### 2.3. Questionnaire Development

The questionnaire utilized in this study was developed based on a comprehensive review of the existing literature on ADHD, aimed at understanding the potential risk factors associated with its development. It adopted a checklist format comprising a series of yes/no, multiple-choice, and short-response items and covered various domains related to demographic and familial characteristics, pregnancy-related factors, birth- and infantile-related factors, as well as ADHD-associated conditions. Demographic and familial characteristics encompassed gender; age; diagnosis age/treatment if ADHD; district of residence; parent’s age at conception; consanguineous marriage; and familial history of ADHD, Autism Spectrum Disorder (ASD), and mental disorders. Pregnancy-related factors encompassed a history of spontaneous abortion, in vitro fertilization, plurality, gestational complications (hypertension, diabetes, pre-eclampsia), anemia, bleeding requiring treatment, maternal stress, physical accidents, dietary habits (coffee consumption, smoking), diseases, infections, and folic acid intake during pregnancy. Birth- and infantile-related factors included the mode of delivery, forceps or vacuum-aided delivery, peri-/postnatal complications, extremely/very premature birth (<32 weeks gestation), birth weight, breastfeeding duration, as well as incidences of child’s head trauma, childhood infections, and adverse experiences. ADHD-associated conditions investigated were migraine, digestive disorders, dyslexia, sleep problems, intellectual disability, self-injurious behaviors, and psychiatric disorders. 

### 2.4. Statistical Analysis

Data were analyzed using SPSS software (Statistical Package for the Social Sciences) version 27.0 (IBM SPSS Inc., Chicago, IL, USA). Descriptive statistics were computed for all variables examined in the study, which included calculating mean and standard deviation (SD) for numerical data, as well as frequencies (*n*) and percentages (%) for categorical data. Categorical variables were compared between the two groups using the Chi-square test or Fisher’s exact test when expected values within cells were below five. For quantitative variables, the independent samples *t*-test was employed to compare means between two groups, after confirming their normal distribution. Data are considered normally distributed when skewness and kurtosis values are between −1 and +1. To control for multiple comparisons, the Benjamani–Hochberg False Discovery Rate (FDR < 5%) adjustment was applied to define statistical significance; the adjusted *p*-value should be lower than 0.05. Finally, a binary logistic regression analysis was conducted, with the presence versus absence of ADHD as the dependent variable and variables that remained significant after the FDR correction in the bivariate analysis as independent variables. Odds ratios (ORs) were also presented with 95% confidence intervals (CIs) to evaluate the variables as potential risk factors.

## 3. Results

### 3.1. Bivariate Analysis

#### 3.1.1. Demographic and Familial Factors

The mean age of the final sample, comprising 119 participants aged 6–24 years, was 13.8 years (±3.4 years). Of these, 61 participants were included in the ADHD group and 58 participants in the control group. Table 1 compares cases and controls for demographic and familial variables. As indicated in Table 1, no significant difference was observed between the groups regarding the districts of residence (*p* = 0.178). In the ADHD group, 31.1% were females and 68.9% were males. For the control group, 34.5% were females, and 65.5% were males. No significant difference in gender distribution between the two groups was observed (*p* = 0.698). ADHD cases had a mean age of 13.3 years (±3.5 years), while controls had a mean age of 14.3 years (±3.3 years). The independent samples *t*-test revealed no statistically significant difference in mean ages between the two groups (*p* = 0.123). For the 61 ADHD patients, the mean age at diagnosis was 8.4 years (±3.7 years). Among these patients, 65.6% were receiving pharmacological treatments at the time of the study. The frequently reported pharmacological treatments included methylphenidate (*n* = 34; 55.7%), followed by risperidone and atomoxetine (*n* = 4; 6.6% each). Valproic acid and clonidine (*n* = 1; 1.6% each) were also mentioned. Examining parental factors, consanguinity did not show a significant association with ADHD in our study (*p* = 0.365). The mean maternal age at conception was significantly higher in the ADHD group (30.6 ± 4.9 years vs. 28.4 ± 4.2 years, *p* = 0.010). After FDR correction, this significant difference persisted (adjusted *p*-value = 0.037). Furthermore, the results revealed a significant association between a familial history of ADHD and ASD and the onset of ADHD. A higher percentage of ADHD patients reported having ADHD patients (32.8% vs. 5.2%, *p* < 0.001) and ASD patients (13.1% vs. 0.0%, *p* = 0.006) in their families compared to controls. After FDR correction, these significances persisted, with an adjusted *p*-value of 0.022 for a family history of ADHD and 0.044 for a family history of ASD. Table 2 illustrates the ADHD-associated conditions observed in the studied ADHD patients. Various conditions were identified, with the most prevalent being digestive disorders (*n* = 24; 39.3%), followed by sleep problems (*n* = 10; 16.4%) and migraine (*n* = 7; 11.5%). Other noted conditions included self-injurious behaviors (*n* = 5; 8.2%), intellectual disability (*n* = 5; 8.2%), dyslexia (*n* = 4; 6.6%), obsessive compulsive disorder (*n* = 2; 3.3%), and oppositional defiant disorder (*n* = 1; 1.6%). 

#### 3.1.2. Pregnancy-Related Factors

Table 3 presents a comparative analysis of pregnancy-related factors between cases and controls. Mothers in the case group demonstrated a significantly greater likelihood of their child being born through in vitro fertilization (18% vs. 3.4%, *p* = 0.011), and a significantly higher prevalence of prenatal anemia (29.5% vs. 12.1%, *p* = 0.020). Additionally, they exhibited a significantly higher frequency of experiencing stress during pregnancy (49.2% vs. 25.9%, *p* = 0.009). After FDR correction, these significances persisted (adjusted *p*-values < 0.05). 

#### 3.1.3. Birth- and Infantile-Related Factors

Table 4 outlines the differences in birth- and infantile-related factors between cases and controls. Cases were more likely to experience C-section delivery (60.7% vs. 39.7%, *p* = 0.022), extremely/very preterm birth (<32 weeks gestation) (13.1% vs. 1.7%, *p* = 0.033), and neonatal jaundice (29.5% vs. 8.6%, *p* = 0.004). A significant difference in breastfeeding was also noted. A higher percentage of ADHD participants were not breastfed compared to the controls (39.3% vs. 19%, *p* = 0.015). Additionally, cases had a significantly higher prevalence of childhood adverse experiences compared to controls (19.7% vs. 3.4%, *p* = 0.006). After applying FDR correction, the significance of the differences remained for all these factors except for prematurity.

### 3.2. Multivariate Analysis

Table 5 represents the results of the logistic regression analysis, with the presence/absence of ADHD as the dependent variable. Breastfeeding (*p* = 0.013, OR = 0.263) was identified as a protective factor against ADHD occurrence, whereas family history of ADHD (*p* < 0.001, OR = 12.033), anemia during pregnancy (*p* = 0.027, OR = 3.654), maternal self-reported stress during pregnancy (*p* = 0.015, OR = 3.268), and neonatal jaundice (*p* = 0.011, OR = 5.020) were significantly associated with an increased risk for ADHD in offspring. However, no association between in vitro fertilization and ADHD was found in this study. 

## 4. Discussion

This pilot study is the first in Lebanon to investigate the correlation between pre-, peri-, and neonatal; psychosocial; and familial factors and ADHD among a sample of Lebanese individuals.

The results of our study showed a male predominance (68.9%) among ADHD patients, consistent with the international literature on ADHD, which reports male-to-female ratios ranging between 2:1 and 10:1 [8]. ADHD affects both males and females, and a commonly proposed hypothesis is that females may be underdiagnosed due to variations in the presentation of the disorder between genders. Females with ADHD manifest less hyperactive/impulsive behaviors and show more inattentive signs than males with the condition. They also tend to show more comorbid internalizing disorders compared to males with ADHD, who present with more externalizing disorders. Thus, females are viewed as less problematic and less apt to be diagnosed with ADHD because of less overt symptoms [8].

Our study displayed a correlation between maternal anemia during pregnancy and ADHD in offspring. In pregnancy, an increased demand for iron arises to augment the maternal red cell mass and support the growth of the fetoplacental unit [32]. Severe maternal anemia can result in fetal and neonatal iron deficiency [33]. According to Al Khatib et al., 16% of women of childbearing age in Lebanon experience anemia, while 27.2% have iron deficiency [34]. As erythrocyte hemoglobin is required for oxygen transport, anemic mothers’ ability to supply oxygen to the developing fetus may be limited, potentially increasing the risk of fetal hypoxia [17]. Moreover, adverse neonatal outcomes associated with prenatal anemia include a low Apgar score, low birth weight, small for gestational age, preterm birth, and increased incidence of cesarean delivery [35]. Neonates with iron deficiency experience cognitive and behavioral impairments, while preclinical research suggests irreversible neurological consequences of prenatal iron deficiency [36]. Iron is essential for crucial developmental processes such as myelination, critical for the speed and accuracy of neuronal communication, cellular differentiation, and the synthesis of monoamine neurotransmitters, which are implicated in the etiology of ADHD [17]. The regulation of these neurotransmitters undergoes significant development in humans from mid-gestation to 3 years postnatal age and preclinical models suggest that early-life iron deficiency not only acutely alters the concentrations of these neurotransmitters and their receptors and reuptake mechanisms, but also has long-term effects persisting into adulthood, even after iron repletion [36].

Furthermore, our investigation identified maternal self-reported stress during pregnancy as a significant risk factor for the development of ADHD in children. In developed nations like Lebanon, several psychosocial and cultural factors contribute to distress during pregnancy. These include a preference for male gender for the baby, unplanned pregnancy, lower income, familial issues with the spouse’s family and parents, marital conflicts, previous spontaneous abortions, and potential complications during pregnancy, all identified as risk factors for prenatal stress in these regions [37,38]. The physiological condition induced by stress during pregnancy can perturb offspring neurodevelopment [39]. One potential mechanism involves increased fetal exposure to cortisol. The placenta, crucial for regulating maternal–fetal interactions, expresses 11β-hydroxysteroid dehydrogenase type 2 (11β-HSD2), an enzyme that converts active cortisol to inactive cortisone, acting as a barrier against elevated glucocorticoids. Studies indicated that maternal stress reduces 11β-HSD2 expression, leading to fetal glucocorticoid overexposure [39]. Elevated cortisol levels can detrimentally affect the fetal hippocampus, leading to reduced neurogenesis and neuronal density [40]. Indeed, maternal stress during pregnancy correlated with a smaller hippocampal volume in newborns, which, in consequence, affected infant social–emotional development [41]. Moreover, exposure to prenatal stress can elevate the likelihood of various adverse outcomes, such as preterm delivery associated with abnormal brain maturation and adverse developmental consequences [42] and shortened telomere length in offspring, an indication of an accelerated life history [43]. 

Moreover, our study identified a significant correlation between neonatal jaundice and the risk of ADHD development in offspring. At high levels, unconjugated bilirubin (UCB) exerts a neurotoxic effect and crosses the blood–brain barrier [44]. Autopsy results of jaundiced neonates revealed scattered yellow spots in most brain regions, with particularly intense coloration in the basal ganglia [45]. Damage to the basal ganglia, which is highly sensitive to bilirubin toxicity, can contribute to ADHD symptoms [46]. Research by Lu et al. investigated abnormalities in brain magnetic resonance imaging (MRI) and brainstem auditory evoked potentials (BAEPs) of neonates with varying bilirubin levels. Their findings revealed that even low levels of bilirubin can cause central nervous system damage observable via MRI and BAEPs, suggesting that subclinical brain injury induced by bilirubin in jaundiced neonates could serve as an early indicator of ADHD risk [47]. Mechanistically, UCB induces neurotoxicity through disruptions in mitochondrial function, ionic imbalance, extracellular glutamate accumulation, glial cell release of inflammatory cytokines, increased production of reactive oxygen species (ROS), and oxidative stress leading to apoptosis [44]. Oxidative stress and neuroinflammation have been shown to contribute to ADHD pathophysiology [48]. Moreover, epigenetic studies indicate that bilirubin neurotoxicity may alter gene expression involved in critical neurobiological processes, such as synaptic plasticity, brain development and differentiation, and neuron maintenance and survival [49]. Understanding these mechanisms underscores the importance of early monitoring and intervention strategies in jaundiced neonates to mitigate long-term ADHD risk.

Our results also revealed a higher prevalence of familial ADHD history within the ADHD group, supporting the influence of genetics on the disorder. Research has demonstrated that the familial aggregation of ADHD increases with increasing genetic relatedness, as reflected in hazard ratios from the study by Chen et al.: 70.45 for monozygotic twins, 8.44 for dizygotic twins, 8.27 for full siblings, 2.86 for maternal half-siblings, 2.31 for paternal half-siblings, 2.24 for full cousins, and 1.47 for half cousins [50]. It is also reported that this aggregation is not solely driven by genetic factors but also by a small contribution from shared environmental factors. Indeed, the higher familial aggregation observed in maternal half-siblings compared to paternal half-siblings suggests the presence of shared environmental influences. Despite having similar genetic sharing, maternal half-siblings tend to share more pregnancy-related environments than paternal half-siblings, including intrauterine and perinatal conditions. Additionally, post parental separation, children often reside with their mothers [50].

In addition to the reported risk factors, our results showed that breastfeeding was associated with a lower risk of developing ADHD, suggesting it may serve as a protective factor. Breast milk provides the essential nutrients an infant requires during the first six months of life, including fats, carbohydrates, proteins, vitamins, minerals, and water. Additionally, it contains bioactive factors, such as cytokines, immunoglobulins, and lysozymes, that enhance the infant’s developing immune system, offering protection against infections [51]. Breast milk is also enriched in miRNAs implicated in gene regulation of dopaminergic/glutamatergic synapses, neurotransmitter secretion, neuron projection morphogenesis, and synaptic vesicle transport [52]. It is possible that breastfeeding is beneficial to the developing brain not just through nutritional effects but also due to mother–infant interactions. Breastfeeding helps develop emotional regulation skills by providing comfort and soothing during distress. The physical closeness, warmth, and nourishment during breastfeeding reduce stress and anxiety while sucking has a calming effect on the baby’s nervous system. Consistent breastfeeding interactions teach infants that their caregiver is a reliable source of comfort, fostering a sense of security and gradually enhancing their ability to self-soothe and regulate emotions, contributing to their emotional resilience and self-regulation skills [53].

Several limitations of our pilot study warrant consideration. Firstly, the sample size could be considered small, yet it remains sufficiently large to ensure adequate statistical power for the analysis. Secondly, the retrospective design of the study results in a low degree of evidence and introduces the possibility of recall bias, as participants were asked to recall information from several years prior. Thirdly, the questionnaire utilized in this investigation had not been previously validated; nevertheless, it was constructed based on findings from the existing literature on ADHD. Additionally, the participants in this study were Lebanese, and thus the findings may not necessarily be transferable to other populations. Moreover, it is worth noting that the presence or absence of correlations investigated in this study may be influenced by the socioeconomic status (SES) of participants and improvements in healthcare practices over the past two decades. The prevalence of factors such as parental age at conception, consanguineous marriage, healthcare access, and exposure to stressors varies with SES, which was not accounted for in this study. Additionally, the broad age range of participants suggests that younger cohorts may benefit from advancements in prenatal and postnatal care, potentially reducing the prevalence of certain factors among younger cohorts compared to older ones. These considerations should be accounted for when interpreting our findings.

Moving forward, future complementary studies should address these limitations by employing larger sample sizes and more precise exposure assessments, such as consulting medical records of obstetric examinations, delivery, and child health; considering a more focused age range; and accounting for potential confounders, which would enhance the robustness of the analysis and provide a more comprehensive understanding of the factors at play in ADHD development. Furthermore, conducting cross-cultural comparisons will provide insights into the broader applicability of findings beyond specific regional contexts.

## 5. Conclusions

ADHD is recognized as one of the most prevalent childhood neurodevelopmental disorders. To date, no studies have been conducted in Lebanon to investigate modifiable correlates among affected individuals, making our study the first of its kind in the country. Therefore, prioritizing epidemiological investigations within the Lebanese population is imperative to achieve conclusive findings. In summary, our study highlights the importance of early screening for maternal anemia during pregnancy, given its association with heightened ADHD risk, alongside nutritional counseling in antenatal care. Moreover, addressing maternal psychological well-being during pregnancy emerges as a potential intervention avenue to mitigate adverse neural and behavioral consequences in offspring. Additionally, our findings underscore the importance of long-term neurologic surveillance and follow-up for children with neonatal jaundice to detect and address ADHD early, thereby improving their overall well-being. Furthermore, our study emphasizes the importance of promoting breastfeeding as a public health strategy to potentially reduce the risk of ADHD and supporting it as a key component of comprehensive early infant care. Lastly, our results suggesting a genetic component underscore the significance of identifying ADHD susceptibility genes. Identification of these genes could lead to specific biological markers for diagnosis and novel therapy development, as well as facilitate early identification of at-risk individuals, enabling prompt treatment implementation to improve patient management. Further investigation into genetic and environmental factors and their interconnection is imperative to expand upon current knowledge and deepen our understanding of the disorder and its associated factors. Subsequent studies analyzing biological samples for genetic, epigenetic, metabolic, and inflammatory markers are necessary to unravel underlying mechanisms and advance research on modifiable risk factors for neurodevelopmental disorders. Moreover, future research should consider longitudinal studies to track the development of children with ADHD over time and gain deeper insights into the long-term effects of the identified risk factors.

## Figures and Tables

**Table 1:** Comparison of demographic and familial characteristics between cases and controls.

Variable	ADHD Group (*n* = 61)	Control Group (*n* = 58)	*X*^2^/*t*	*df*	*p*-Value	Adjusted *p*-Value
Gender			0.150	1	0.698 (*X*^2^)	
Female	19 (31.1%)	20 (34.5%)				
Male	42 (68.9%)	38 (65.5%)				
District of residence			6.299	4	0.178 (*X*^2^)	
North	14 (23%)	16 (27.6%)				
Bekaa	3 (4.9%)	5 (8.6%)				
Mount Lebanon	37 (60.7%)	23 (39.7%)				
Beirut	6 (9.8%)	11 (19%)				
South	1 (1.6%)	3 (5.2%)				
Age (years)			−1.555	117	0.123 (*t*)	
mean ± SD	13.3 ± 3.5	14.3 ± 3.3				
ADHD medications			57.290	1	<*0.001* (*X*^2^)	
Yes	40 (65.6%)	0 (0%)				
No	21 (34.4%)	58 (100%)				
Mother’s age at conception (years) mean ± SD	30.6 ± 4.9	28.4 ± 4.2	2.625	117	*0.010* (*t*)	**0.037**
Consanguineous marriage			1.725	1	0.365 (*F)*	0.502
Yes	4 (6.6%)	1 (1.7%)				
No	57 (93.4%)	57 (98.3%)				
Family history of ADHD ^1^			14.540	1	<*0.001* (*X*^2^)	**0.022**
Yes	20 (32.8%)	3 (5.2%)				
No	41 (67.2%)	55 (94.8%)				
Family history of ASD ^1^			8.155	1	*0.006* (*F*)	**0.044**
Yes	8 (13.1%)	0 (0.0%)				
No	53 (86.9%)	58 (100.0%)				

^1^ Family history was considered in first-degree, second-degree, and third-degree relatives. *X*^2^, Pearson Chi-square test; *t*, Independent samples *t*-test; *F*, Fisher’s exact test. Italicizing indicates *p*-value < 0.05. Bolding indicates statistically significant after FDR correction (adjusted *p*-value < 0.05).

**Table 2:** ADHD-associated conditions.

Variable	ADHD Group (*n* = 61)	Control Group (*n* = 58)
Migraine		
Yes	7 (11.5%)	1 (1.7%)
No	54 (88.5%)	57 (98.3%)
Digestive disorders ^1^		
Yes	24 (39.3%)	11 (19%)
No	37 (60.7%)	47 (81%)
Obsessive compulsive disorder		
Yes	2 (3.3%)	0 (0%)
No	59 (96.7%)	58 (100%)
Oppositional defiant disorder		
Yes	1 (1.6%)	0 (0%)
No	60 (98.4%)	58 (100%)
Self-injurious behaviors		
Yes	5 (8.2%)	0 (0%)
No	56 (91.8%)	58 (100%)
Dyslexia		
Yes	4 (6.6%)	0 (0%)
No	57 (93.4%)	58 (100%)
Sleep problems ^2^		
Yes	10 (16.4%)	0 (0%)
No	51 (83.6%)	58 (100%)
Intellectual disability		
Yes	5 (8.2%)	0 (0%)
No	56 (91.8%)	58 (100%)

^1^ Digestive disorders included constipation, diarrhea, stomach ache, nausea, and gastroesophageal reflux. ^2^ Sleep problems encompassed subjective descriptions of challenges affecting sleep quality, such as sleep-onset difficulties, fragmented sleep, and disruptions in the sleep–wake cycle.

**Table 3:** Comparison of pregnancy-related factors between cases and controls.

Variable	ADHD Group (*n* = 61)	Control Group (*n* = 58)	*X* ^2^	*df*	*p*-Value	Adjusted *p*-Value
In vitro fertilization			6.499	1	*0.011* (*X*^2^)	**0.035**
Yes	11 (18%)	2 (3.4%)				
No	50 (82%)	56 (96.6%)				
Plurality			1.935	1	0.273 (*F*)	0.400
Singleton	55 (90.2%)	56 (96.6%)				
Multiple (twin or triplet)	6 (9.8%)	2 (3.4%)				
Gestational hypertension			0.396	1	0.612 (*F*)	0.709
Yes	1 (1.6%)	2 (3.4%)				
No	60 (98.4%)	56 (96.6%)				
Gestational diabetes			2.926	1	0.244 (*F*)	0.383
Yes	3 (4.9%)	0 (0%)				
No	58 (95.1%)	58 (100%)				
Pre-eclampsia			0.959	1	1.000 (*F*)	1
Yes	1 (1.6%)	0 (0%)				
No	60 (98.4%)	58 (100%)				
Anemia during pregnancy			5.449	1	*0.020* (*X*^2^)	**0.049**
Yes	18 (29.5%)	7 (12.1%)				
No	43 (70.5%)	51 (87.9%)				
Maternal self-reported stress during pregnancy ^1^			6.875	1	*0.009* (*X*^2^)	**0.040**
Yes	30 (49.2%)	15 (25.9%)				
No	31 (50.8%)	43 (74.1%)				
Coffee consumption during pregnancy			0.457	2	0.796 (*X*^2^)	0.876
Nondrinker	28 (45.9%)	28 (48.3%)				
≤14 cups/week	18 (29.5%)	14 (24.1%)				
>14 cups/week	15 (24.6%)	16 (27.6%)				
Smoking during pregnancy			0.743	1	0.389 (*X*^2^)	0.503
Yes	7 (11.5%)	4 (6.9%)				
No	54 (88.5%)	54 (93.1%)				
Folic acid intake during pregnancy			0.042	1	0.838 (*X*^2^)	0.878
Yes	41 (67.2%)	40 (69%)				
No	20 (32.8%)	18 (31%)				
Infections during pregnancy ^2^			0.559	1	0.455 (*X*^2^)	0.556
Yes	24 (39.3%)	19 (32.8%)				
No	37 (60.7%)	39 (67.2%)				

^1^ Stress during pregnancy was reported to be caused by factors such as death or severe disease of a relative, war, family or marital problems, financial difficulties, partner’s death, and work-related stress. ^2^ Reported infections during pregnancy included seasonal flu, urinary tract infections, vaginal fungal infections, food poisoning, sinus infection, toxoplasmosis, and an instance of elevated C-reactive protein levels. *X*^2^, Pearson Chi-square test; *F*, Fisher’s exact test. Italicizing indicates *p*-value < 0.05. Bolding indicates statistically significant after FDR correction (adjusted *p*-value < 0.05).

**Table 4:** Comparison of birth- and infantile-related factors between cases and controls.

Variable	ADHD Group (*n* = 61)	Control Group (*n* = 58)	*X* ^2^	*df*	*p*-Value	Adjusted *p*-Value
Mode of delivery			5.245	1	*0.022* (*X*^2^)	**0.048**
Vaginal	24 (39.3%)	35 (60.3%)				
C-section	37 (60.7%)	23 (39.7%)				
Lack of oxygen at birth			2.602	1	0.208 (*F*)	0.381
Yes	5 (8.2%)	1 (1.7%)				
No	56 (91.8%)	57 (98.3%)				
Extremely/very preterm birth (<32 weeks gestation)			5.518	1	*0.033* (*F*)	0.066
Yes	8 (13.1%)	1 (1.7%)				
No	53 (86.9%)	57 (98.3%)				
Neonatal jaundice			8.319	1	*0.004* (*X*^2^)	**0.044**
Yes	18 (29.5%)	5 (8.6%)				
No	43 (70.5%)	53 (91.4%)				
Breast-feeding			5.947	1	*0.015* (*X*^2^)	**0.041**
Yes	37 (60.7%)	47 (81%)				
No	24 (39.3%)	11 (19%)				
Childhood infections ^1^			1.427	1	0.232 (*X*^2^)	0.393
Yes	14 (23%)	19 (32.8%)				
No	47 (77%)	39 (67.2%)				
Childhood adverse experiences ^2^			7.539	1	*0.006* (*X*^2^)	**0.033**
Yes	12 (19.7%)	2 (3.4%)				
No	49 (80.3%)	56 (96.6%)				

^1^ Reported childhood infections included roseola, measles, mumps, chickenpox, rotavirus, bronchitis, pneumonia, urticaria, bacterial gastroenteritis, viral-induced asthma, staphylococcal skin infection, and ear infections. ^2^ Childhood adverse experiences comprised difficult situations, such as parental divorce, loss of a family member, exposure to physical violence, emotional neglect, bullying, and family dysfunction. *X*^2^, Pearson Chi-square test; *F*, Fisher’s exact test. Italicizing indicates *p*-value < 0.05. Bolding indicates statistically significant after FDR correction (adjusted *p*-value < 0.05).

**Table 5:** Logistic regression analysis taking the presence/absence of ADHD as the dependent variable.

Variable	*p*-Value	OR	95% CI
Family history of ADHD	**<0.001**	12.033	2.950–49.072
In vitro fertilization	0.064	5.135	0.909–29.011
Anemia during pregnancy	**0.027**	3.654	1.158–11.529
Maternal self-reported stress during pregnancy	**0.015**	3.268	1.263–8.456
Neonatal jaundice	**0.011**	5.020	1.438–17.532
Breastfeeding	**0.013**	0.263	0.092–0.757

Variables entered in the model: family history of ADHD, in vitro fertilization, anemia during pregnancy, maternal self-reported stress during pregnancy, neonatal jaundice, and breastfeeding. Bolding indicates *p*-value < 0.05.

## Data Availability

Dataset available on request from the authors.
